# *trans*-Tetra­aqua­bis­(pyridazine-4-car­box­yl­ato-κ*O*)magnesium(II) dihydrate

**DOI:** 10.1107/S1600536811004168

**Published:** 2011-02-09

**Authors:** Wojciech Starosta, Janusz Leciejewicz

**Affiliations:** a Institute of Nuclear Chemistry and Technology, ul. Dorodna 16, 03-195 Warszawa, Poland

## Abstract

The crystal structure of the title compound, [Mg(C_5_H_3_N_2_O_2_)_2_(H_2_O)_4_]·2H_2_O, is composed of centrosymmetric monomers in which an Mg^II^ ion is coordinated by two carboxyl­ate O atoms from the two pyridazine-4-carboxylate ligands. The monomers linked by O—H⋯O and O—H⋯N hydrogen bonds into layers which are held together by hydrogen bonds in which solvent water O atoms act as donors and acceptors, resulting in a three-dimensional network.

## Related literature

For the crystal structure of a Pb(II) complex with pyridazine-4-carboxyl­ate and water ligands, see: Starosta & Leciejewicz, (2009 ▶). The structure of pyridazine-4-carb­oxy­lic acid hydro­chloride was determined earlier (Starosta & Leciejewicz, 2008 ▶). The structure of a Mg^II^ complex with pyridazine-3-carboxyl­ate and water ligands has been also reported by Gryz *et al.* (2006 ▶). 
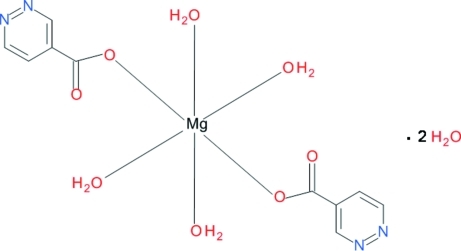


## Experimental

### 

#### Crystal data

[Mg(C_5_H_3_N_2_O_2_)_2_(H_2_O)_4_]·2H_2_O
*M* *_r_* = 378.59
Monoclinic, 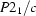

*a* = 7.2571 (15) Å
*b* = 11.688 (2) Å
*c* = 10.550 (2) Å
β = 108.36 (3)°
*V* = 849.3 (3) Å^3^
*Z* = 2
Mo *K*α radiation
μ = 0.16 mm^−1^
*T* = 293 K
0.24 × 0.22 × 0.08 mm

#### Data collection

Kuma KM-4 four-circle diffractometer
Absorption correction: analytical (*CrysAlis RED*; Oxford Diffraction, 2008) ▶ *T*_min_ = 0.968, *T*_max_ = 0.987
2007 measured reflections
1873 independent reflections
1136 reflections with *I* > 2σ(*I*)
*R*_int_ = 0.023
3 standard reflections every 200 reflections  intensity decay: 1.3%

#### Refinement

*R*[*F* ^2^ > 2σ(*F* ^2^)] = 0.036
*wR*(*F* ^2^) = 0.122
*S* = 1.04
1873 reflections
139 parameters
6 restraints
H atoms treated by a mixture of independent and constrained refinement
Δρ_max_ = 0.28 e Å^−3^
Δρ_min_ = −0.21 e Å^−3^

### 

Data collection: *KM-4 Software* (Kuma, 1996 ▶); cell refinement: *KM-4 Software*; data reduction: *DATAPROC* (Kuma, 2001 ▶); program(s) used to solve structure: *SHELXS97* (Sheldrick, 2008 ▶); program(s) used to refine structure: *SHELXL97* (Sheldrick, 2008 ▶); molecular graphics: *SHELXTL* (Sheldrick, 2008 ▶); software used to prepare material for publication: *SHELXTL*.

## Supplementary Material

Crystal structure: contains datablocks I, global. DOI: 10.1107/S1600536811004168/kp2307sup1.cif

Structure factors: contains datablocks I. DOI: 10.1107/S1600536811004168/kp2307Isup2.hkl

Additional supplementary materials:  crystallographic information; 3D view; checkCIF report

## Figures and Tables

**Table 1:** Hydrogen-bond geometry (Å, °)

*D*—H⋯*A*	*D*—H	H⋯*A*	*D*⋯*A*	*D*—H⋯*A*
O3—H31⋯O5	0.83 (2)	1.97 (2)	2.775 (3)	164 (3)
O4—H42⋯N1^i^	0.81 (2)	2.01 (2)	2.817 (2)	172 (3)
O5—H51⋯N2^ii^	0.82 (2)	1.98 (2)	2.798 (3)	179 (3)
O3—H32⋯O2^iii^	0.80 (2)	1.92 (2)	2.675 (2)	159 (3)
O5—H52⋯O2^iv^	0.81 (2)	1.97 (2)	2.765 (3)	168 (3)
O4—H41⋯O5^v^	0.80 (2)	1.97 (2)	2.766 (3)	175 (3)

Symmetry codes: (i) 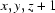; (ii) 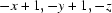; (iii) 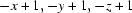; (iv) 
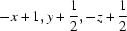
; (v) 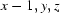.

**Fig. 1.**
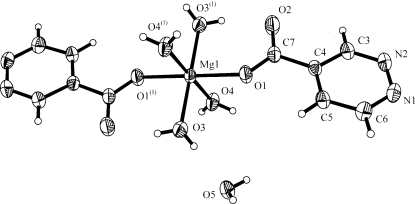
A structural unit of (I) with atom labelling scheme and the 50% probability displacement ellipsoids. Symmetry code: (i) -x + 1,-y + 1,-z + 1. (ii) x, y, z + 1.

**Fig. 2.**
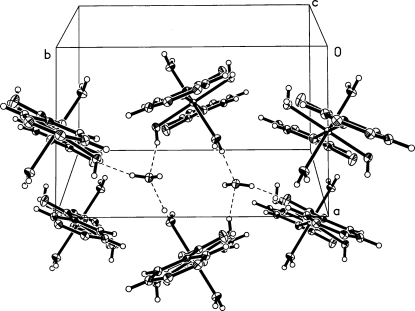
Crystal packing of I.

## supplementary crystallographic information

### Comment

The structure of the title compound (I) is built of monomeric molecules in which the Mg^+2^ located in an inversion centre is chelated by two carboxylate atoms each donated by one of symmetry ralated ligand molecules and by two pairs of aqua O atoms resulting in a slightly distorted octahedral geometry. The carboxylate O1, O1^(i)^ and aqua O3, O3^(i)^ atoms form an equatorial plane, aqua O4 and O4^(i)^ atoms are at the axial positions. The observed Mg—O bond lengths and bond angles are almost the same as reported for the complex with pyridazine-3-carboxylate and water ligands (Gryz *et al.*, 2006). The pyridazine ring is planar with r.m.s. of 0.0046 (1) Å. The observed bond distances and angles are close to those reported for the parent acid (Starosta & Leciejewicz, 2008). The carboxylate group is rotated from the mean plane by 8.1 (1)°. Hydrogen bonds link the monomers to form molecular sheets. They operate between coordinated water O atoms as donors and uncoordinated carboxylate O atoms and pyridazine-N atoms in adjacent monomers as acceptors. The sheets are held together by hydrogen bonds in which crystal water molecules act as donors and acceptors resulting in a three-dimensional network. The coordination mode reported in the structure of a Mg^II^ complex with pyridazine-3-carboxylate and water ligands is also octahedral but the Mg^II^ ion is coordinated by a pair of symmetry related N,*O*-chelating groups of the ligands and a pair of water O atoms (Gryz *et al.*, 2006). The Pb(II) complex with the title ligand shows entirely different coordination mode. Two symmetry related metal ions form a dimer in which they are bridged by hetero-ring N atoms of two symmetry related ligands amd two aqua-O atoms. Each Pb(II) ion is also coordinated by both carboxylate O atoms of another ligand whose hetero-ring N atoms do not coordinate to Pb(II). (Starosta & Leciejewicz, 2009).

### Experimental

The title compound was obtained by mixing boiling aqueous solutions, one containig 2 mmols of pyridazine-4-carboxylic acid (Aldrich), the other 1 mmol of magnesium diacetate tetrahydrate (Aldrich). The mixture was boiled under reflux for two h, then cooled to room temperature and left to crystallise. A few days latter, colourless crystalline plates were found after evaporation to dryness. They were recrystallised from water several times until well formed single crystals were obtained. Crystals were washed with cold ethanol and dried in the air.

### Refinement

Water hydrogen atoms were located in a difference map and were allowed to ride on the parent atom with *U*_iso_(H)=1.5*U*_eq_(O). H atoms attached to pyridazine-ring C atoms were positioned at calculated positions and were treated as riding on the parent atoms, with C—H=0.93 Å and *U*_iso_(H)=1.5*U*_eq_(C).

### Crystal data

**Table:**

[Mg(C_5_H_3_N_2_O_2_)_2_(H2O)_4_]·2H2O	*F*(000) = 396
*M**_r_* = 378.59	*D*_x_ = 1.480 Mg m^−^^3^
Monoclinic, *P*2_1_/*c*	Mo *K*α radiation, λ = 0.71073 Å
*a* = 7.2571 (15) Å	Cell parameters from 25 reflections
*b* = 11.688 (2) Å	θ = 6–15°
*c* = 10.550 (2) Å	µ = 0.16 mm^−^^1^
β = 108.36 (3)°	*T* = 293 K
*V* = 849.3 (3) Å^3^	Plate, colourless
*Z* = 2	0.24 × 0.22 × 0.08 mm

### Data collection

**Table:**

Kuma KM-4 four-circle diffractometer	1136 reflections with *I* > 2σ(*I*)
Radiation source: fine-focus sealed tube	*R*_int_ = 0.023
graphite	θ_max_ = 27.7°, θ_min_ = 2.7°
profile data from ω/2θ scans	*h* = −9→0
Absorption correction: analytical (*CrysAlis RED*; Oxford Diffraction, 2008)	*k* = 0→15
*T*_min_ = 0.968, *T*_max_ = 0.987	*l* = −13→12
2007 measured reflections	3 standard reflections every 200 reflections
1873 independent reflections	intensity decay: 1.3%

### Refinement

**Table:**

Refinement on *F*^2^	Primary atom site location: structure-invariant direct methods
Least-squares matrix: full	Secondary atom site location: difference Fourier map
*R*[*F*^2^ > 2σ(*F*^2^)] = 0.036	Hydrogen site location: inferred from neighbouring sites
*wR*(*F*^2^) = 0.122	H atoms treated by a mixture of independent and constrained refinement
*S* = 1.04	*w* = 1/[σ^2^(*F*_o_^2^) + (0.0628*P*)^2^ + 0.293*P*] where *P* = (*F*_o_^2^ + 2*F*_c_^2^)/3
1873 reflections	(Δ/σ)_max_ < 0.001
139 parameters	Δρ_max_ = 0.28 e Å^−^^3^
6 restraints	Δρ_min_ = −0.21 e Å^−^^3^

### Special details

**Table:**

Geometry. All e.s.d.'s (except the e.s.d. in the dihedral angle between two l.s. planes) are estimated using the full covariance matrix. The cell e.s.d.'s are taken into account individually in the estimation of e.s.d.'s in distances, angles and torsion angles; correlations between e.s.d.'s in cell parameters are only used when they are defined by crystal symmetry. An approximate (isotropic) treatment of cell e.s.d.'s is used for estimating e.s.d.'s involving l.s. planes.
Refinement. Refinement of *F*^2^ against ALL reflections. The weighted *R*-factor *wR* and goodness of fit *S* are based on *F*^2^, conventional *R*-factors *R* are based on *F*, with *F* set to zero for negative *F*^2^. The threshold expression of *F*^2^ > σ(*F*^2^) is used only for calculating *R*-factors(gt) *etc*. and is not relevant to the choice of reflections for refinement. *R*-factors based on *F*^2^ are statistically about twice as large as those based on *F*, and *R*- factors based on ALL data will be even larger.

### Fractional atomic coordinates and isotropic or equivalent isotropic displacement parameters (Å^2^)

**Table:**

	*x*	*y*	*z*	*U*_iso_*/*U*_eq_	
Mg1	0.5000	0.5000	0.5000	0.0236 (3)	
O1	0.3810 (2)	0.54000 (14)	0.29844 (14)	0.0318 (4)	
O2	0.2156 (3)	0.38329 (15)	0.20927 (16)	0.0453 (5)	
N1	0.2554 (3)	0.58217 (18)	−0.19230 (18)	0.0339 (5)	
C7	0.2921 (3)	0.47760 (19)	0.2019 (2)	0.0284 (5)	
N2	0.1945 (3)	0.47789 (17)	−0.16963 (18)	0.0336 (5)	
C5	0.3386 (3)	0.62622 (19)	0.0395 (2)	0.0311 (5)	
H5	0.3895	0.6782	0.1084	0.037*	
C4	0.2765 (3)	0.52021 (19)	0.0639 (2)	0.0260 (5)	
C3	0.2036 (3)	0.4491 (2)	−0.0472 (2)	0.0312 (5)	
H3	0.1587	0.3770	−0.0338	0.037*	
C6	0.3231 (4)	0.6534 (2)	−0.0916 (2)	0.0344 (5)	
H6	0.3626	0.7257	−0.1092	0.041*	
O4	0.2870 (2)	0.59132 (16)	0.54856 (16)	0.0347 (4)	
O5	0.9316 (3)	0.67116 (16)	0.38743 (16)	0.0358 (4)	
O3	0.6662 (3)	0.64863 (14)	0.52618 (17)	0.0338 (4)	
H31	0.751 (4)	0.642 (3)	0.489 (3)	0.056 (10)*	
H42	0.267 (5)	0.586 (3)	0.620 (2)	0.058 (10)*	
H51	0.896 (4)	0.628 (2)	0.323 (2)	0.041 (8)*	
H32	0.711 (5)	0.656 (3)	0.6052 (19)	0.062 (10)*	
H52	0.905 (5)	0.7357 (18)	0.360 (3)	0.065 (11)*	
H41	0.187 (3)	0.614 (2)	0.498 (3)	0.048 (9)*	

### Atomic displacement parameters (Å^2^)

**Table:**

	*U*^11^	*U*^22^	*U*^33^	*U*^12^	*U*^13^	*U*^23^
Mg1	0.0276 (5)	0.0249 (5)	0.0176 (5)	−0.0002 (4)	0.0061 (4)	0.0006 (4)
O1	0.0389 (9)	0.0329 (8)	0.0207 (7)	−0.0020 (7)	0.0052 (6)	−0.0005 (6)
O2	0.0681 (13)	0.0384 (10)	0.0276 (8)	−0.0184 (9)	0.0126 (8)	−0.0026 (7)
N1	0.0380 (11)	0.0409 (11)	0.0239 (9)	0.0057 (9)	0.0111 (8)	0.0042 (8)
C7	0.0290 (11)	0.0335 (12)	0.0221 (10)	0.0032 (9)	0.0073 (9)	−0.0013 (8)
N2	0.0374 (11)	0.0386 (11)	0.0221 (9)	0.0020 (8)	0.0056 (8)	−0.0031 (7)
C5	0.0363 (12)	0.0314 (12)	0.0242 (10)	0.0000 (9)	0.0077 (9)	−0.0046 (9)
C4	0.0223 (10)	0.0325 (12)	0.0221 (10)	0.0044 (8)	0.0052 (8)	−0.0014 (8)
C3	0.0353 (12)	0.0309 (12)	0.0252 (11)	0.0003 (10)	0.0063 (9)	−0.0028 (9)
C6	0.0408 (13)	0.0328 (12)	0.0305 (11)	0.0024 (10)	0.0125 (10)	0.0013 (10)
O4	0.0337 (9)	0.0476 (10)	0.0230 (8)	0.0114 (8)	0.0091 (7)	0.0051 (7)
O5	0.0403 (10)	0.0352 (10)	0.0269 (8)	0.0016 (8)	0.0035 (7)	−0.0006 (7)
O3	0.0407 (10)	0.0342 (9)	0.0276 (9)	−0.0055 (7)	0.0123 (8)	0.0010 (7)

### Geometric parameters (Å, °)

**Table:**

Mg1—O4^i^	2.0714 (17)	C5—C4	1.370 (3)
Mg1—O4	2.0714 (17)	C5—C6	1.389 (3)
Mg1—O1^i^	2.0807 (16)	C5—H5	0.9300
Mg1—O1	2.0807 (16)	C4—C3	1.398 (3)
Mg1—O3	2.0829 (17)	C3—H3	0.9300
Mg1—O3^i^	2.0829 (17)	C6—H6	0.9300
Mg1—H32	2.42 (3)	O4—H42	0.809 (18)
O1—C7	1.254 (3)	O4—H41	0.798 (18)
O2—C7	1.248 (3)	O5—H51	0.819 (17)
N1—C6	1.317 (3)	O5—H52	0.809 (18)
N1—N2	1.343 (3)	O3—H31	0.828 (18)
C7—C4	1.509 (3)	O3—H32	0.799 (18)
N2—C3	1.316 (3)		
			
O4^i^—Mg1—O4	180.00 (6)	O2—C7—O1	126.0 (2)
O4^i^—Mg1—O1^i^	92.02 (7)	O2—C7—C4	116.88 (19)
O4—Mg1—O1^i^	87.99 (7)	O1—C7—C4	117.1 (2)
O4^i^—Mg1—O1	87.98 (7)	C3—N2—N1	119.28 (19)
O4—Mg1—O1	92.02 (7)	C4—C5—C6	117.8 (2)
O1^i^—Mg1—O1	180.0	C4—C5—H5	121.1
O4^i^—Mg1—O3	90.95 (7)	C6—C5—H5	121.1
O4—Mg1—O3	89.05 (7)	C5—C4—C3	116.1 (2)
O1^i^—Mg1—O3	90.88 (7)	C5—C4—C7	123.40 (19)
O1—Mg1—O3	89.12 (7)	C3—C4—C7	120.4 (2)
O4^i^—Mg1—O3^i^	89.05 (7)	N2—C3—C4	124.0 (2)
O4—Mg1—O3^i^	90.95 (7)	N2—C3—H3	118.0
O1^i^—Mg1—O3^i^	89.12 (7)	C4—C3—H3	118.0
O1—Mg1—O3^i^	90.88 (7)	N1—C6—C5	123.5 (2)
O3—Mg1—O3^i^	180.000 (1)	N1—C6—H6	118.3
O4^i^—Mg1—H32	95.0 (8)	C5—C6—H6	118.3
O4—Mg1—H32	85.0 (8)	Mg1—O4—H42	123 (2)
O1^i^—Mg1—H32	72.6 (6)	Mg1—O4—H41	127 (2)
O1—Mg1—H32	107.4 (6)	H42—O4—H41	105 (3)
O3—Mg1—H32	18.6 (6)	H51—O5—H52	108 (3)
O3^i^—Mg1—H32	161.4 (6)	Mg1—O3—H31	110 (2)
C7—O1—Mg1	129.89 (15)	Mg1—O3—H32	105 (2)
C6—N1—N2	119.35 (19)	H31—O3—H32	112 (3)

Symmetry codes: (i) −*x*+1, −*y*+1, −*z*+1.

### Hydrogen-bond geometry (Å, °)

**Table:**

*D*—H···*A*	*D*—H	H···*A*	*D*···*A*	*D*—H···*A*
O3—H31···O5	0.83 (2)	1.97 (2)	2.775 (3)	164 (3)
O4—H42···N1^ii^	0.81 (2)	2.01 (2)	2.817 (2)	172 (3)
O5—H51···N2^iii^	0.82 (2)	1.98 (2)	2.798 (3)	179 (3)
O3—H32···O2^i^	0.80 (2)	1.92 (2)	2.675 (2)	159 (3)
O5—H52···O2^iv^	0.81 (2)	1.97 (2)	2.765 (3)	168 (3)
O4—H41···O5^v^	0.80 (2)	1.97 (2)	2.766 (3)	175 (3)

Symmetry codes: (ii) *x*, *y*, *z*+1; (iii) −*x*+1, −*y*+1, −*z*; (i) −*x*+1, −*y*+1, −*z*+1; (iv) −*x*+1, *y*+1/2, −*z*+1/2; (v) *x*−1, *y*, *z*.
